# Model of human epidermis reconstructed *in vitro* with keratinocytes and melanocytes on dead de-epidermized human dermis

**DOI:** 10.1590/S1516-31802004000100006

**Published:** 2004-01-08

**Authors:** Rehder Jussara, Souto Luís Ricardo Martinhão, Issa Cláudia Maria Bernardino Magro, Maria Beatriz Puzzi

**Keywords:** Epidermis, Melanocyte, Culture, Keratinocyte, Epiderme, Cultura, Melanócitos, Queratinócitos, Cultura de celulas.

## Abstract

**CONTEXT:**

Recent progress in the field of epithelial culture techniques has allowed the development of culture systems in which the reconstructed epidermis presents characteristics of morphological differentiation similar to those seen *in vivo*. Human epidermis reconstructed *in vitro* may be used as the best alternative for the *in vitro* testing of the toxicology and efficiency of products for topical use, as well as in the treatment of skin burns and chronic skin ulcers.

**OBJECTIVE:**

To demonstrate a method for obtaining human epidermis reconstructed *in vitro*, using keratinocytes and melanocytes cultivated on dead de-epidermized human dermis.

**TYPE OF STUDY:**

Experimental/laboratory.

**SETTING:**

Skin Cell Culture Laboratory of the Faculdade de Ciências Médicas, Universidade Estadual de Campinas, Campinas, São Paulo, Brazil.

**PROCEDURE:**

Human keratinocytes and melanocytes cultured *in vitro* were grown on a biological matrix (dead de-epidermized human dermis) and the system was kept at an air-liquid interface, in a suitable culturing medium, until a stratified human epidermis was formed, maintaining the histological characteristics of the epidermis *in vivo*.

**RESULTS:**

It was histologically demonstrated that it is possible to reproduce a differentiated epidermis through keratinocytes and melanocytes cultured on dead de-epidermized human dermis, thus obtaining a correctly positioned human epidermis reconstructed *in vitro* with functional keratinocytes and melanocytes that is similar to *in vivo* epidermis.

**CONCLUSIONS:**

It is possible to obtain a completely differentiated human epidermis reconstructed *in vitro* from keratinocyte and melanocyte cultures on a dead de-epidermized human dermis.

## RESUMO

**CONTEXTO:**

Recentes progressos no campo das técnicas de cultura epitelial têm levado ao desenvolvimento de sistemas de cultura nos quais a epiderme reconstruída obtida exibe características de diferenciação morfológica semelhantes àquelas vistas *in vivo*. Uma epiderme humana reconstruída *in vitro* pode ser utilizada como melhor alternativa para testes toxicológicos e de eficácia de produtos de uso tópico *in vitro* e ainda no tratamento de queimaduras e úlceras crônicas de pele.

**OBJETIVO:**

Demonstrar um método de obtenção de epiderme humana reconstruída *in vitro*, utilizando queratinócitos e melanócitos cultivados sobre uma derme humana morta desepidermizada.

**TIPO DE ESTUDO:**

Experimental laboratorial.

**LOCAL:**

Laboratório de Cultura de Células da Pele da Faculdade de Ciências Médicas da Universidade Estadual de Campinas, Campinas, São Paulo, Brasil.

**PROCEDIMENTOS:**

Queratinócitos e melanócitos humanos cultivados *in vitro* foram semeados sobre uma matriz biológica (derme humana morta desepidermizada) e o sistema foi mantido em interface ar-líquido, em meio de cultura adequado, até haver a formação de uma epiderme humana estratificada, mantendo as características histológicas da epiderme *in vivo*.

**RESULTADOS:**

Demonstramos, histologicamente, que é possível reproduzir uma epiderme diferenciada, a partir da cultura de queratinócitos e melanócitos sobre uma derme humana morta desepidermizada, obtendo uma epiderme humana reconstruída in *vitro*, com queratinócitos e melanócitos funcionais, corretamente posicionados, equivalente à epiderme *in vivo*.

**CONCLUSÕES:**

É possível obter uma epiderme humana reconstruída *in vitro* completamente diferenciada a partir da cultura de queratinócitos e melanócitos sobre uma derme humana morta desepidermizada.

## INTRODUCTION

Epidermal differentiation is a process in which keratinocytes are morphologically and biochemically modified. Leaving the *stratum basale*, they move through the *stratum spinosum* and *stratum granulosum* and stop at the upper layer (*stratum corneum*), thus constituting multilamellar structures of anucleated corneocytes surrounded by extracellular lipids. In addition to the keratinocytes, the basal membrane contains melanocytes, which are cells responsible for pigmenting the skin, with the synthesis of melanin that is progressively transferred to the keratinocytes.^1-3^

The dermis is composed of a dense tissue of collagen and elastic fibers produced by dermal fibroblasts, which provides the physical consistency of the skin. It contains blood and lymph vessels as well as nerves, which inform the organism about its interaction with the environment. It also contains hair follicles, sweat and sebaceous glands.^4^

Degeneration of dermal and epidermal elements may occur in extensive, deep skin and mucosal lesions, without spontaneous tissue regeneration. In such cases it is possible to use autologous or allogenic transplants of frozen or lyophilized human or animal skin, synthetic tissues or biodegradable materials.^1^

An option for the *in vitro* culturing of autologous cells has recently emerged, with the aim of regenerating the destroyed cutaneous tegument. Through technological advances in epithelial cell culturing, models of the epidermis reconstructed *in vitro* have been achieved, presenting characteristics of morphological and biochemical differentiation similar to those seen *in vivo*.^5-9^

Over the last few years, several laboratories have made continuous efforts to obtain living skin models *in vitro*, so as to investigate the regulation of keratinocyte proliferation and differentiation and for efficacy tests on toxicology and skin products.^10,11^

The method for keratinocyte culturing at the air-liquid interface was first described by Pruniéras et al. in 1983.^12^ Currently, several methods are available.^5-10,12-14^

At the Skin Cell Culture Laboratory of Faculdade de Ciências Médicas, Universidade Estadual de Campinas, the method for keratinocyte and melanocyte culturing and achievement of reconstructed epidermis at the air-liquid interface, which was developed by Pruniéras et al. and improved by Bessou et al. in 1995,15 has been modified, implemented and improved, with the aim of obtaining a reconstructed epidermis equivalent to *in vivo* epidermis.

## MATERIAL AND METHODS

### Collection of material

Skin fragments from patients submitted to breast and abdomen surgical procedures at the University of Campinas Teaching Hospital were collected. This procedure was in accordance with the ethical standards of the Ethics Committee of Faculdade de Ciências Médicas, Universidade Estadual de Campinas, Campinas, São Paulo, Brazil.

### Preparation of culture samples

The material was placed in sterile tempered glass jars and conserved in 0.9% physiological serum refrigerated to 4° C, without exceeding a limit of 12 hours until its manipulation.

The skin fragments were separated from the adipose tissue, placed on a Petri dish (Corning) and sectioned into pieces of 2 to 3 mm, using a surgical instrument under a laminar flow culture hood, so as to keep the whole procedure sterile.

These fragments were placed on a new Petri dish with 10 ml of 0.25% trypsin solution and 1 mM of ethylenediamine tetraacetic acid (GIBCO BRL, Grand Island, New York, USA, cat. no. 25200-056), with the epidermis always facing upwards. They were then incubated in an oven at 37° C, with 5% CO_2_ tension for four hours. This procedure resulted in separation of the epidermis from the dermis.

After this period, the trypsin was neutralized using the same volume of fetal bovine serum (GIBCO, cat. no. 10270-106) and the suspension was filtered in a 50 ml tube (Falcon) with a 40-mm nylon filter (Falcon code 2340).

This suspension was centrifuged at 1,200 rpm and 4° C for 10 minutes and the supernatant was discarded, thus obtaining a cell "pool" containing keratinocytes and melanocytes, which were resuspended in 5 ml of 0.9% saline solution. After this, one aliquot was removed for manual cell counting in a Neubauer chamber using the trypan blue exclusion method.

### Cell culturing

The cells were divided among Corning culture flasks, with 1 × 105 cells per cm^2^ and incubated at 37° C, with 5% CO_2_ tension, in a specific culture medium for keratinocytes and melanocytes.

#### Culture medium for keratinocytes

**Figure 1:**
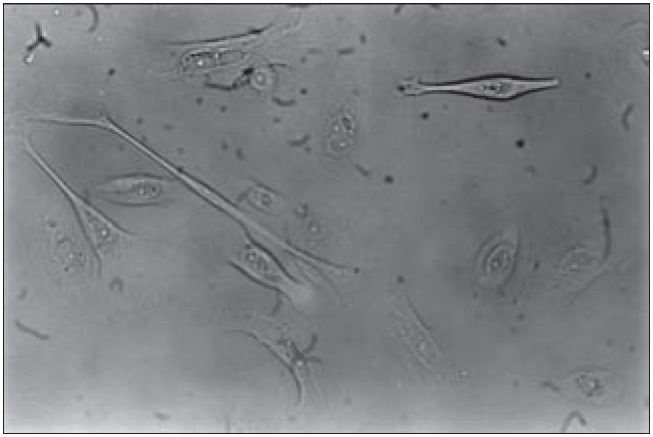
Primary keratinocyte culture. Inverted microscopy (200 X).

Keratinocyte culture medium was used (GIBCO cat. no. 10724-011), complemented with L-glutamine 2 mM/ml, penicillin 100 UI/ml, streptomycin 0.1 mg/ml (GIBCO cat. no. 10378-016) and fetal bovine serum 10%. Cell adhesion to the culture flasks occurred within 48 hours, thus obtaining the primary keratinocyte culture (Figure 1).

#### Culture medium for melanocytes

Melanocyte culture medium MCDB 153 was used (Sigma Chemical Co., St. Louis, Missouri, USA, M 7403), complemented with L-glutamine 2 mM/ml, penicillin 100 UI/ml, streptomycin 0.1 mg/ml, fetal bovine serum 10%, epidermis growth factor 5 µg/ml (GIBCO cat. no. 10450-013), bovine pituitary extract 50 µg/ml (GIBCO cat. no. 13028-014), hydrocortisone 0.6 µg/ml (Sigma H 0888) and bovine insulin 3 µg/ml (GIBCO cat. no. 13007-018).

**Figure 2:**
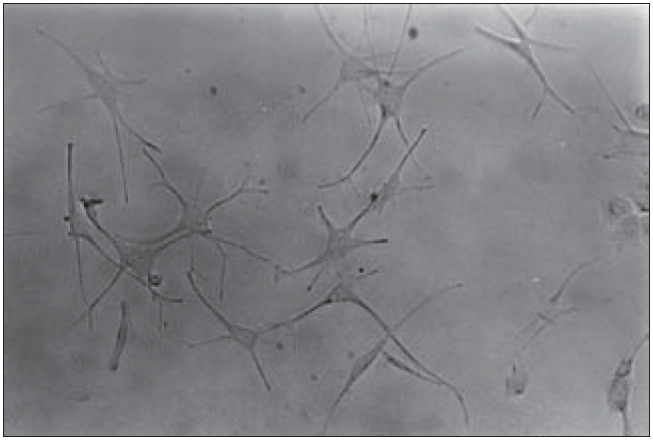
Primary melanocyte culture. Inverted microscopy (200 X).

Cell adhesion to the culture flasks occurred within 48 hours, thus obtaining the primary melanocyte culture (Figure 2).

**Figure 3:**
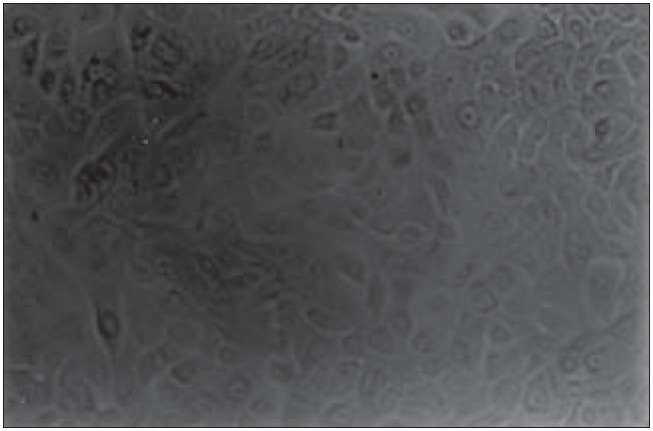
Confluent keratinocyte culture. Inverted microscopy (200 X).

The culture medium (for keratinocytes and melanocytes) was changed every three days. When the flask wall was totally covered by cells (Figure 3), we cut them into small pieces.

### Preparation of the dead de-epidermized human dermis

In order to obtain the reconstructed epidermis *in vitro*, melanocytes and keratinocytes need to be reproduced on a substrate. For this, we chose to use dermis, which we named dead de-epidermized human dermis, following the technique described by Pruniéras et al. (1979).^16,17^

The skin originated from patients submitted to corrective breast and abdomen surgery at the University of Campinas Teaching Hospital, it was cut into fragments of 2.0 × 2.0 cm. The skin squares were rinsed in 70° GL alcohol and then put in 0.9% saline solution with antibiotics (penicillin 100 UI/ml, streptomycin 0.1 mg/ml), and incubated for 10 days at 37° C. Then the epidermis was separated from the dermis.

### Developing reconstructed epidermis

The keratinocyte and melanocyte cultures were prepared separately (centrifuged), to be seeded on the de-epidermized dermis. The melanocyte to keratinocyte ratio used was 1:40.

The dead de-epidermized human dermis was placed on a grid and/or gauze and the mixed epidermal cells were seeded with 2 × 106 cells per cm² on the dermis, in 150 ul of keratinocyte culture medium contained by a polypropylene ring. Then this seeded dermis was incubated at 37° C, with 5% CO_2_ tension for 48 hours, which was the time needed for cell adhesion to the dermis.

After this period the polypropylene rings were removed and the system (dermis plus cells) was submersed in epidermis culture medium.

#### Culture medium for epidermis

Three parts of Iscove's Modified Dulbecco's Medium (IMDM — GIBCO cat. no. 12200-036) and one part of keratinocyte culture medium (GIBCO cat. no. 10724-011) were used, complemented with L-glutamine 2 mM/ml, penicillin 100 UI/ml, streptomycin 0.1 mg/ml (GIBCO cat. no. 10378-016) and fetal bovine serum 10%.

Seventy-two hours later, the system was maintained at the air-liquid interface and the medium was complemented with Ca++ 1.5 mM and kept for 20 days, with three weekly changes.

### Morphological studies of reconstructed human epidermis *in vitro*

**Figure 4:**
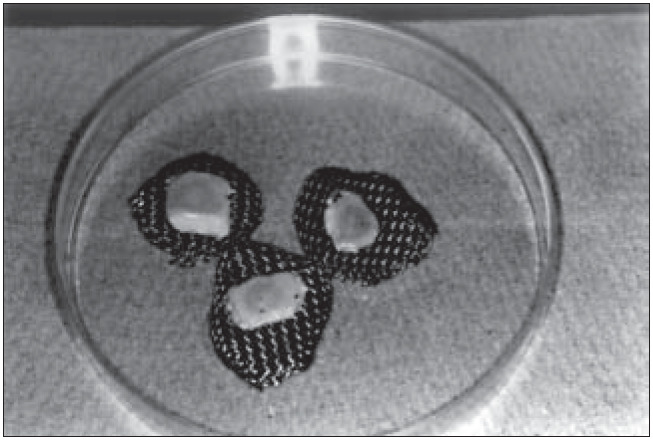
Dead de-epidermized human dermis with epidermis reconstructed on steel grids.

The system was interrupted after being maintained at the air-liquid interface for 20 days (Figure 4), fixed in formaldehyde 10% and paraffin-embedded. Histological cuts colored with hematoxylin-eosin (HE) were made.

## RESULTS

**Figure 5:**
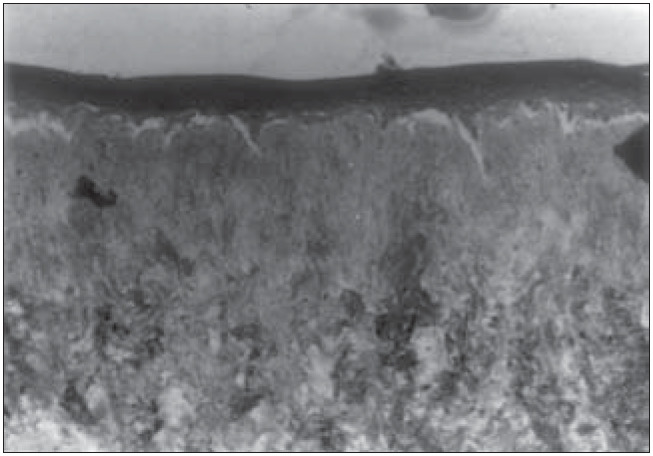
Human epidermis reconstructed in vitro on dead de-epidermized human dermis. Optical microscopy. Hematoxylin-eosin (HE) staining (165 X).

**Figure 6:**
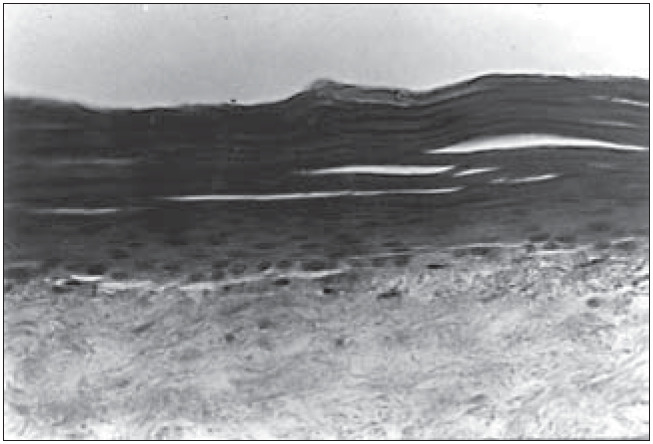
Human epidermis reconstructed in vitro on dead de-epidermized human dermis. Optical microscopy. Hematoxylin-eosin (HE) staining (330 X).

**Figure 7:**
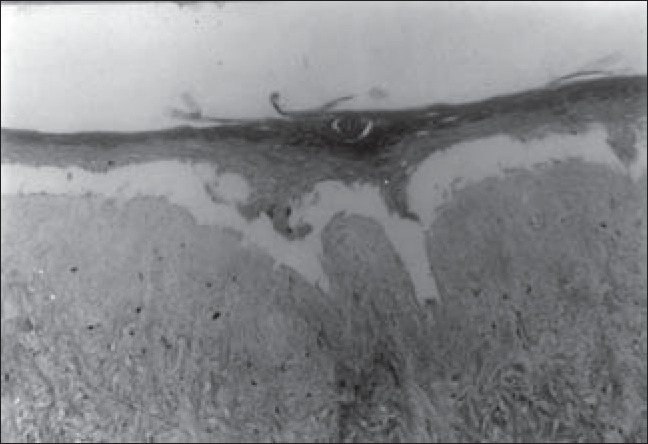
Epidermis reconstructed in vitro, in the process of separation from the dead de-epidermized human dermis. Optical microscopy. Hematoxylin-eosin staining (165 X).

**Figure 8:**
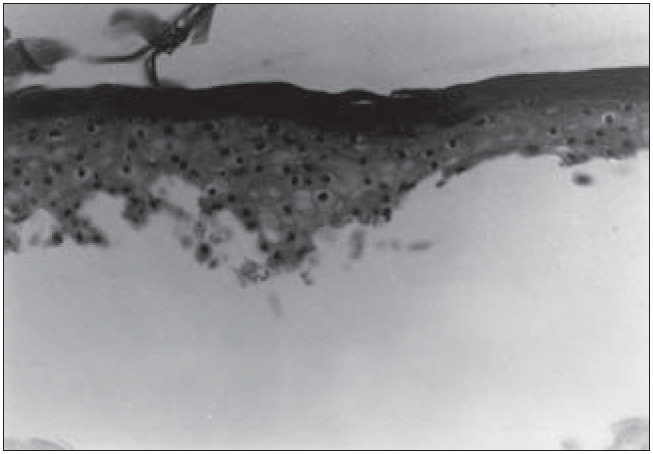
Epidermis reconstructed in vitro, in the process of separation from the dead de-epidermized human dermis. Optical microscopy. Hematoxylin-eosin (HE) staining (330 X).

We were able to histologically demonstrate, through the hematoxylin-eosin (HE) staining, that it is possible to reproduce a completely differentiated epidermis reconstructed *in vitro* from keratinocyte and melanocyte cultures on a dead de-epidermized human dermis (Figures 5 and 6), with functional keratinocytes and melanocytes that are correctly positioned, equivalent to epidermis *in vivo*. The extent of the stratification and keratinization of human epidermis reconstructed *in vitro* had the same characteristics as found *in vivo* (Figures 7 and 8).

After developing the human epidermis reconstructed *in vitro*, we successfully triplicated the experiment to validate the technique.

## DISCUSSION

The present study, although it describes a method that is sophisticated and difficult to put into practice, showed that it is possible to obtain a model of reconstructed human epidermis using materials and methodology different from those previously described, with the purpose of enabling laboratory investigations and clinical treatments that have been difficult to obtain in our country up to the present day.

Over the course of a two-year period, we had attempted to reproduce in its totality the technique used by foreign authors. We did not obtain cell reproduction in the cultures until we standardized the addition of fetal bovine serum 10% directly to the melanocyte and keratinocyte culture media.

The model of human epidermis reconstructed *in vitro* provides a good system for studies, especially in relation to tests on the efficiency and toxicology of new chemicals and drugs *in vitro*.^10,11^

Ultraviolet rays affect epidermal differentiation. Therefore, it is possible to study the effects of solar radiation on an epidermis composed of melanocytes and keratinocytes. This model does not allow the study of the immunological effects of radiation measured by Langerhans cells or UV-induced macrophages.^15^ However, it does allow the study of the biological effects of irradiation, particularly lipid peroxidation.^3^ It also allows us to study the effect of sunscreens to validate the photoprotection model (non-immunological).

This model will allow us to study the physiopathology and possible therapies for still-undetermined pigmentary affections such as vitiligo, melasma and the formation of melanocytic nevus.

The transplantation of cultured autologous keratinocytes is the most advanced area of tissue engineering and it has an important application in the restoration of skin lesions such as burns and chronic ulcers.^18^ The reconstructed epidermis is physiologically compatible with autografts.^9,14,18^

The use of autografts is limited by the extent of the donor site and the clinical condition of patients, in the case of large lesions. Allotransplants collected from cadavers or volunteers are rejected after one or two weeks and provide only temporary cover. Human or animal skin grafts that are devitalized, lyophilized or refrigerated in glycerol accommodate the connective tissue and stimulate blood vessel growth, but in general are prematurely degraded. The treatment of large skin lesions with reconstructed autologous epidermis offers an attractive alternative to replace existing therapies since, from a small skin fragment of the patient, we can obtain cell cultures that multiply rapidly and can be cryopreserved, thereby allowing their use for new treatments for an indeterminate time and making the removal of new skin fragments unnecessary.^1^

The real challenge in the twenty-first century will be to reproduce the whole skin. In fact, our interest in the present study was only the epidermis. It would be interesting to introduce the Langerhans cells into this model that is already quite advanced. Such a procedure would have the objective of restoring the immune function to the skin.^7^

The utilization of this model on dead deepidermized human dermis facilitates the adhesion of keratinocyte and melanocyte through the preservation of the basal membrane constituents.^19^ However, it would also be interesting to reproduce this system on a more physiological dermis. The types of dermis for such a proposal have not yet been well developed.

The model of human epidermis reconstructed *in vitro* presented herein has low prospects for clinical use in burns and chronic skin ulcers. This is not only because of the difficulty in removing the reconstructed epidermis from dead de-epidermized human dermis without causing lesions, but also because it does not present an associated dermis. Otherwise, as already mentioned, it possesses excellent applicability for laboratory studies.

## CONCLUSION

It is possible to obtain a sufficient number of cells from human keratinocyte and melanocyte cultures for emplacement in dead deepidermized human dermis. This allows the formation of a completely differentiated human epidermis reconstructed *in vitro*.

Our next step would be to improve this system, with the purpose of reproducing human dermis with viable fibroblasts inside it, in order to facilitate the adhesion, multiplication and differentiation of the epidermal cells, and to clinically use such dermis in association with this epidermis.
